# Enrollment in HIV Care Two Years after HIV Diagnosis in the Kingdom of Swaziland: An Evaluation of a National Program of New Linkage Procedures

**DOI:** 10.1371/journal.pone.0150086

**Published:** 2016-02-24

**Authors:** Duncan A. MacKellar, Daniel Williams, Nosipho Storer, Velephi Okello, Charles Azih, Jennifer Drummond, Harriet Nuwagaba-Biribonwoha, Peter Preko, Rebecca L. Morgan, Makhosazana Dlamini, Johnita Byrd, Simon Agolory, Andrew L. Baughman, Margaret L. McNairy, Ruben Sahabo, Peter Ehrenkranz

**Affiliations:** 1 Division of Global HIV/AIDS, National Center for Global Health, Centers for Disease Control and Prevention, Atlanta, Georgia, United States of America; 2 ICAP at Columbia University, Mbabane, Swaziland; 3 Swaziland National AIDS Programme, Swaziland Ministry of Health, Mbabane, Swaziland; 4 ICAP at Columbia University, New York, New York, United States of America; 5 CTS Global assigned to Centers for Disease Control and Prevention Country Office, Mbabane, Swaziland; 6 McMaster University, Hamilton, Ontario, Canada; 7 Population Services International Country Program, Mbabane, Swaziland; 8 ICF International, Atlanta, Georgia, United States of America; 9 Bill & Melinda Gates Foundation, Seattle, Washington, United States of America; University of Malaya, MALAYSIA

## Abstract

To improve early enrollment in HIV care, the Swaziland Ministry of Health implemented new linkage procedures for persons HIV diagnosed during the Soka Uncobe male circumcision campaign (SOKA, 2011–2012) and the Swaziland HIV Incidence Measurement Survey (SHIMS, 2011). Abstraction of clinical records and telephone interviews of a retrospective cohort of HIV-diagnosed SOKA and SHIMS clients were conducted in 2013–2014 to evaluate compliance with new linkage procedures and enrollment in HIV care at 92 facilities throughout Swaziland. Of 1,105 clients evaluated, within 3, 12, and 24 months of diagnosis, an estimated 14.0%, 24.3%, and 37.0% enrolled in HIV care, respectively, after adjusting for lost to follow-up and non-response. Kaplan-Meier functions indicated lower enrollment probability among clients 14–24 (*P* = 0.0001) and 25–29 (*P* = 0.001) years of age compared with clients >35 years of age. At 69 facilities to which clients were referred for HIV care, compliance with new linkage procedures was low: referral forms were located for less than half (46.8%) of the clients, and few (9.6%) were recorded in the appointment register or called either before (0.3%) or after (4.9%) their appointment. Of over one thousand clients newly HIV diagnosed in Swaziland in 2011 and 2012, few received linkage services in accordance with national procedures and most had not enrolled in HIV care two years after their diagnosis. Our findings are a call to action to improve linkage services and early enrollment in HIV care in Swaziland.

## Introduction

The considerable benefits of antiretroviral therapy (ART) to reduce HIV-related morbidity and mortality, and HIV transmission risk to uninfected sexual partners, are now well established [1–3]. Mathematical models suggest high ART coverage in communities might also substantially reduce HIV incidence [4,5]. These real and plausible benefits, however, cannot be fully realized without high coverage of HIV testing and counseling (HTC), early enrollment in care following HIV diagnosis, and initiation and retention in ART care [1–5]. Although many HIV-infected persons do not enroll early in care following diagnosis, high enrollment rates following diagnosis are possible, particularly among persons who receive evidence-based linkage interventions or who live close to or who already attend health care [1,6–8,9–16]. Local and national programs designed to improve enrollment in care for all HIV-infected persons, however, often lack the resources to implement recommended interventions. The need to evaluate real-world linkage-to-care programs is particularly important for countries with high HIV morbidity and for programs that target groups known to be at high risk for low enrollment in HIV care.

Of all countries, the Kingdom of Swaziland has the highest reported HIV prevalence (32%) among adults 18–49 years of age [17]. Notably, more than half of patients in ART care in Swaziland are estimated to initiate ART late in the course of their disease [18]. While many factors contribute to late initiation of ART, delay in enrollment in care following HIV diagnosis is thought to be particularly important [1,6–8]. To help improve early enrollment in HIV care, in 2010 the Swaziland Ministry of Health expanded HIV care in many rural clinics as part of a national effort to decentralize care, and in 2011, implemented a new set of linkage and retention standard operating procedures (Linkage SOP) [19]. The Linkage SOP was implemented first for clients newly HIV diagnosed through provider-initiated HTC conducted as part of Soka Uncobe (SOKA), a national voluntary male medical circumcision (VMMC) campaign in 2011 and 2012, and through home-based HTC conducted as part of the Swaziland HIV Incidence Measurement Survey (SHIMS) in 2011 [17,20]. Providing linkage services for these two populations was particularly important because young men and persons diagnosed at home can be at exceptionally high risk for delaying enrollment in care [16,21–28].

To evaluate the Swaziland Linkage SOP, we conducted a retrospective enrollment-in-care study among HIV diagnosed SOKA and SHIMS clients who were supposed to have received Linkage SOP services. Most prior linkage studies have assessed enrollment in care at only one or a few facilities over short post-diagnostic periods (e.g., ≤ 6 months)—methods that have limited generalizability and that are reasonably expected to underestimate enrollment in HIV care [6,7]. In our study, we assessed compliance with the Linkage SOP and enrollment in HIV care at 92 facilities throughout Swaziland for up to two years after diagnosis. Because HIV care was largely decentralized at the time of SOKA and SHIMS, we also evaluated enrollment-in-care differences between small clinics and larger hospitals and health centers located within both rural and urban areas of Swaziland.

## Materials and Methods

### Linkage Services

In accordance with the Linkage SOP, a triplicate-copy HTC form was used to prompt linkage services delivered by staff at HIV-care facilities to which clients were referred (referral facilities) [19]. Unique patient identifiers (e.g., full name and date of birth), consent for follow-up contact, telephone number, HIV test results, referral facility, and date expected at the referral facility (typically two weeks after diagnosis) were recorded on the triplicate form. The white copy of the form was provided to clients who were instructed to present the form at first visit to the referral facility, the pink copy was sent via courier to the referral facility, and the yellow copy was retained by the HTC provider [19]. After receipt of the pink copy, staff at referral facilities were expected to: (1) store the pink copy of the HTC form in an "expected-in" binder, (2) record referred clients in the appointment register on the date of expected arrival, (3) review the appointment register and call or text appointment reminders to clients *before* their appointment date (if consent and telephone number were recorded), and (4) call clients 3 days *after* their missed appointment to assess and resolve barriers to care (if applicable) [19]. Clients who consented for follow-up contact who could not be reached by phone could also be visited at home by a nurse or healthcare worker. For clients who visited the facility, the white copy of the HTC form (if presented) was expected to be attached and stored with the pink copy in a separate "arrived patient" binder [19]. Excluding private facilities, training on the Linkage SOP was conducted at all HIV-care facilities during SHIMS and SOKA.

### Study Population

The study population included clients newly HIV-diagnosed through (1) home-based HTC conducted as part of SHIMS from February 1, 2011 through June 30, 2011, and (2) provider-initiated HTC conducted at 13 VMMC sites as part of SOKA from March 1, 2011 through March 31, 2012. SHIMS was a nationally representative sample of domiciled adult residents [17]. The 13 SOKA sites were selected of 31 total sites because of resource constraints and because they accounted for 89% of all clients who tested HIV-positive during Soka Uncobe [20]. Clients of these two populations were initially eligible for the retrospective-cohort study if their archived HTC forms (1) had complete information on date of test, full name, date of birth, HIV testing history, current test result, and referral facility, and (2) indicated the client had tested HIV-positive, and had never previously tested for HIV or had last tested HIV-negative. For SOKA clients, eligibility was further restricted to male clients referred to facilities with >2 referrals. This threshold was imposed because insufficient resources were available to visit all facilities to which only one or two clients were referred. A referral threshold was not imposed on SHIMS clients to achieve a protocol-required 1:1 ratio of male SOKA and SHIMS clients.

### Data Collection and Management

Standard forms were used to (1) record HIV facility characteristics (e.g., type and location of facility); (2) document the availability of data sources at each facility (e.g., electronic medical records and paper registers); (3) abstract clinical data from available sources (e.g., dates of visit and CD4 count); and (4) to interview clients who were not initially verified to have enrolled in care. At the time of this study, Swaziland national treatment guidelines recommended ART at World Health Organization (WHO) clinical stage III or IV, or at CD4 < 350 cells/μl regardless of clinical stage [29]. The interview questionnaire included measures on the name of the referral facility, enrollment in HIV care at referral or non-referral (alternate) facilities, linkage-services received, and reasons for enrolling in care at an alternate facility or not enrolling in care at all (if applicable).

Abstraction teams visited both referral and alternate facilities to verify enrollment in care from 18 September 2013 through 11 February 2014. Teams used the HIV chronic-care file (medical chart) as the primary data source to match client identifiers on the HTC form and to abstract clinical data. If the medical chart could not be located, data were abstracted from other matching facility records (e.g., electronic database or register). Standard methods were routinely used to identify enrolled clients including querying the electronic medical record system (if available), interviewing counselors or nurses, searching stored HTC forms and medical charts, and reviewing appointment, pre-ART, and ART registers from the date of HIV diagnosis through the date of abstraction. To review registers, data abstractors worked in teams of two searching for no more than two names at a time. Field supervisors recorded the availability of data sources, and conducted brief interviews with a medical officer or in-charge nurse to record facility characteristics.

Trained study interviewers called clients who were not initially verified to have enrolled in care at the referral facility and on whom consent and a telephone number was documented on the HTC form. Once contacted, interviewers verified the client’s identity, read a standard consent statement, and administered the questionnaire if the client consented. If clients reported enrolling in care, data-abstraction teams visited those facilities, verified enrollment, and abstracted data in accordance with the above procedures. Clients who reported never enrolling in care were encouraged to enroll and were provided referral appointments when requested.

All data forms were reviewed for completeness and logic inconsistences, and were returned to staff for corrections or re-abstraction when needed. Facility audits were led by senior investigators at study mid- and end-points to assess the accuracy of matching and data abstraction, and to correct abstractions when needed. All study forms were double-data entered in CSPro 5.0 (U.S. Census Bureau), and data analyses were conducted using SAS 9.3 (SAS Institute Inc., Cary, NC, USA).

### Outcome Measures and Analysis

Verified enrollment in HIV care was defined as documentation of either WHO clinical staging or having received HIV services at least once *after* the date a CD4 test was conducted. Clients who were CD4 tested but who did not return for their results were not defined as “enrolled” because it was standard policy at many clinics to draw blood for CD4 testing as the *only* service on the first visit. For each study-gender group (SHIMS female, SHIMS male, SOKA male), findings are reported as percentages for nominal and ordinal variables, and as medians and interquartile ranges (Q1–Q3) for ratio-scaled variables. Group differences were evaluated with the chi-squared (χ^2^) and t-test (*t*) statistics for categorical and ratio-scaled variables, respectively. Kaplan-Meier analyses were conducted to evaluate time to verified enrollment in HIV care and the log-rank (*LR*) test was used to evaluate differences in survival functions. Figures displaying enrollment-in-care functions were restricted to the 800-day period following HIV diagnosis. Clients who were determined not to have enrolled in HIV care were censored; time to censoring was defined as the interval in days from date of HIV diagnosis to the date of medical-record abstraction and determination of non-enrollment. Because all SHIMS participants were HIV diagnosed before 1 July 2011, no SHIMS participants were censored in the 800-day period following diagnosis. For all analyses, p-values (*P*) < 0.05 were considered statistically significant.

Finally, because many clients who were not initially verified as having enrolled in care were lost to follow-up and not interviewed, we estimated the number of clients who were likely to enroll in care by applying the age-group-specific, verified enrollment-in-care probabilities of interviewed clients to the corresponding age-group distribution of clients who were not verified to have enrolled in care and were not interviewed. This procedure was used only to estimate enrollment-in-care in our sample of clients and was not used for Kaplan-Meier and other statistical analyses.

### Human Subjects

The study protocol was reviewed and approved by institutional review boards (IRBs) in Swaziland, and at Columbia University, and the United States Centers for Disease Control and Prevention. In accordance with 45 Code of Federal Regulations 46.116, IRBs granted (1) a waiver for obtaining informed consent to abstract HIV program and clinical data (abstraction), and (2) a waiver for obtaining written informed consent to conduct telephone interviews. These waivers were granted because the evaluation (1) did not involve human-subjects interactions (abstraction only); (2) involved no more than minimal risk to clients; (3) would not adversely affect the rights and welfare of clients; and (4) could not be carried out without the waiver because it was not possible to obtain consent of all eligible clients for data abstraction and written consent of clients contacted for telephone interviews. Before administering telephone interviews, a standardized script was routinely used to obtain verbal informed consent. Verbal consent was documented on the interview screening form and entered into the electronic database.

## Results

### Sample Description

**Fig 1:**
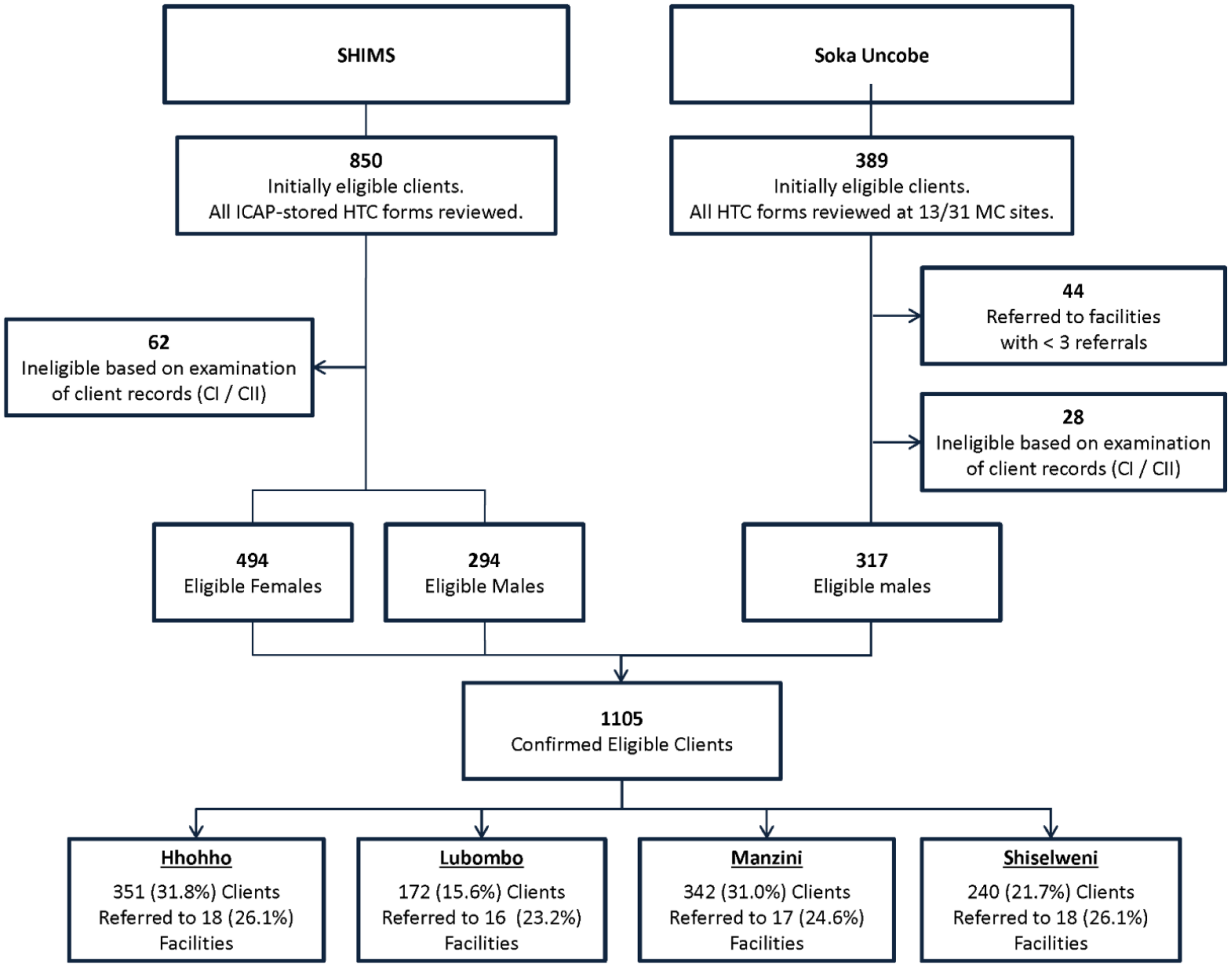
Distribution of newly HIV diagnosed SHIMS and SOKA clients referred to health facilities in the four regions of Swaziland.

**Fig 2:**
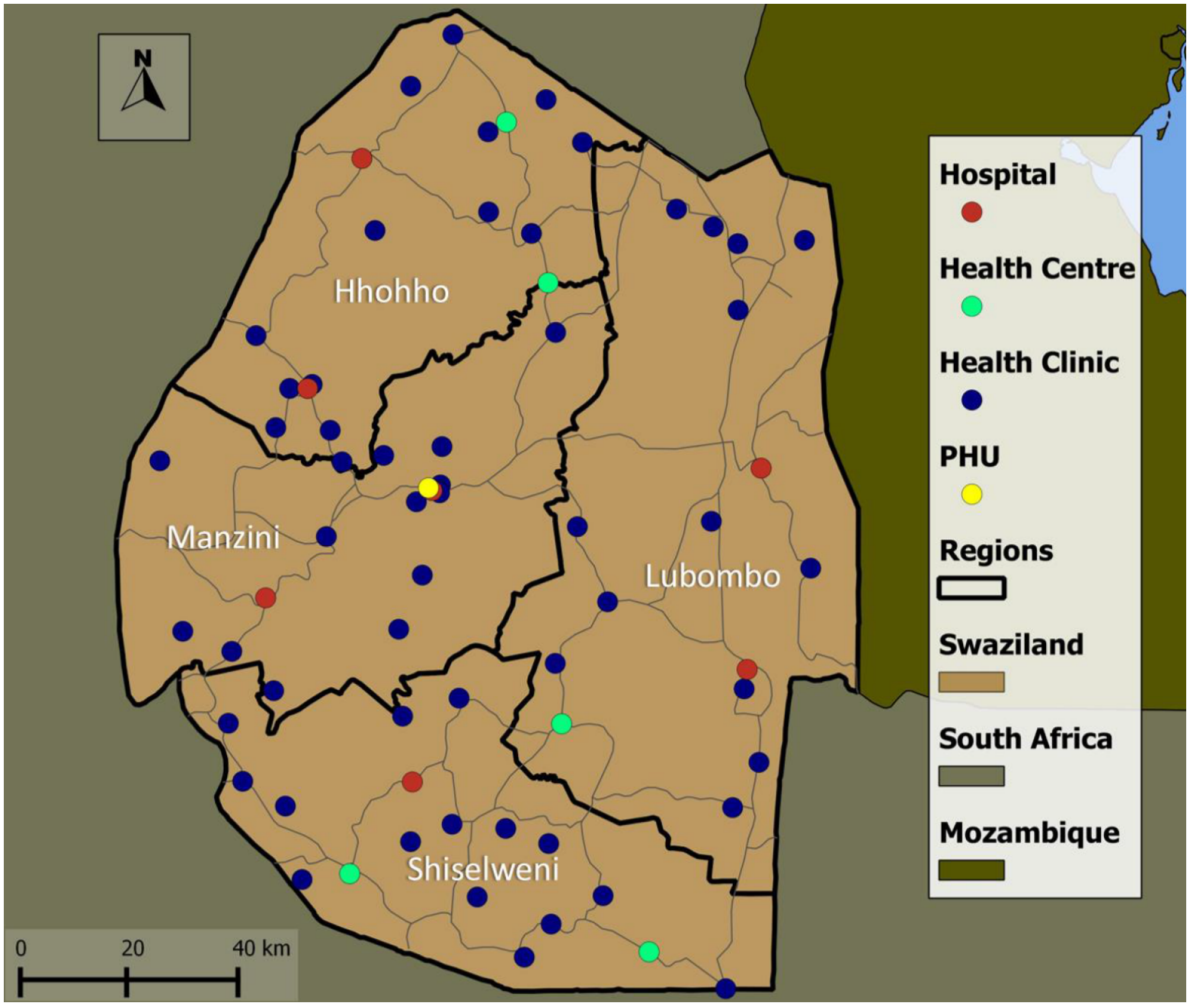
Distribution of 69 HIV care facilities to which eligible SHIMS and SOKA clients were referred at HIV diagnosis, by class of facility.

Review of archived SOKA HTC forms identified 389 initially eligible male clients (Fig 1). Of these, 44 were referred to HIV-care facilities that received only one or two referrals and were excluded from the study. Of the remaining 345 clients, 28 (8.1%) were identified at facilities to have been in HIV care before their SOKA HIV test date and were excluded from analyses (Fig 1). Review of archived forms at ICAP-Swaziland identified 850 initially eligible SHIMS clients, of whom 62 (7.3%) were identified at facilities to have been in HIV care before their SHIMS HIV test date and were excluded from analyses (Fig 1). The combined analytic sample of 1,105 SHIMS (n = 788) and SOKA (n = 317) clients were referred to 69 facilities located throughout Swaziland’s four regions (Fig 2).

### Client and Referral Facility Characteristics

Of the 1,105 eligible clients, the median (Q1–Q3) age at HIV diagnosis was 29 years (24–35); SHIMS females were younger than SHIMS males (*t* = -8.44; *P* < 0.0001) and SOKA males (*t* = -4.46; *P* < 0.0001) (Table 1). At the time of their diagnosis, clients were predominantly referred to government-operated facilities (71.4%), and facilities classified as clinics (47.4%) and hospitals (34.4%); 14.7% of clients were referred to facilities operated by private or non-government organizations, or the Swaziland military. Compared with SHIMS clients, SOKA clients were more likely to be referred to a non-governmental organization (NGO) or private facility (χ^2^ = 140.7; *P* < 0.0001), an urban facility (χ^2^ = 60.1; *P* < 0.0001), and a facility located on a paved road (χ^2^ = 43.3; *P* < 0.0001) (Table 1). All clients tested in either setting were referred to facilities that provided ART refills on site (100%), and nearly all were referred to facilities that initiated ART (98.6%), and that had phones (95.6%), monthly airtime credit [median (Q1–Q3) Swaziland emalangeni: 150 (150–200)], and staff designated to call clients in accordance with the Linkage SOP (Table 1).

**Table 1:** Clisssent and referral facility characteristics, by study-gender group.

Characteristic	SHIMS Female (N = 494)	SHIMS Male (N = 294)	SOKA Male (N = 317)	All Clients (N = 1105)
Age at diagnosis, median (Q1–Q3)	26 (22–33)	32 (27–38)	29 (25–35)	29 (24–35)
Age at diagnosis (years)				
<25	204 (41.3%)	37 (12.6%)	58 (18.3%)	299 (27.1%)
25–29	122 (24.7%)	72 (24.5%)	105 (33.1%)	299 (27.1%)
30–35	81 (16.4%)	84 (28.6%)	87 (27.4%)	252 (22.8%)
>35	87 (17.6%)	101 (34.4%)	67 (21.1%)	255 (23.1%)
Region of referral facility				
Hhohho	148 (30.0%)	101 (34.4%)	102 (32.2%)	351 (31.8%)
Lubombo	83 (16.8%)	51 (17.3%)	38 (12.0%)	172 (15.6%)
Manzini	135 (27.3%)	81 (27.6%)	126 (39.7%)	342 (31.0%)
Shiselweni	128 (25.9%)	61 (20.7%)	51 (16.1%)	240 (21.7%)
Type of referral facility				
Government (non-military)	369 (74.7%)	231 (78.6%)	189 (59.6%)	789 (71.4%)
Faith-based	91 (18.4%)	39 (13.3%)	23 (7.3%)	153 (13.8%)
Private	24 (4.9%)	15 (5.1%)	46 (14.5%)	85 (7.7%)
Non-governmental Organization	8 (1.6%)	4 (1.4%)	46 (14.5%)	58 (5.2%)
Military	2 (0.4%)	5 (1.7%)	13 (4.1%)	20 (1.8%)
Class of referral facility				
Hospital	166 (33.6%)	109 (37.1%)	105 (33.1%)	380 (34.4%)
Health Center	92 (18.6%)	43 (14.6%)	36 (11.4%)	171 (15.5%)
Clinic	227 (46.0%)	137 (46.6%)	160 (50.5%)	524 (47.4%)
Public Health Unit	9 (1.8%)	5 (1.7%)	16 (5.0%)	30 (2.7%)
Location of referral facility				
Urban	176 (35.6%)	110 (37.4%)	188 (59.3%)	474 (42.9%)
Peri-urban	66 (13.4%)	56 (19.0%)	45 (14.2%)	167 (15.1%)
Rural	252 (51.0%)	128 (43.5%)	84 (26.5%)	464 (42.0%)
Referral facility on a paved road	370 (74.9%)	217 (73.8%)	292 (92.1%)	879 (79.5%)
Days per week HIV services provided				
Monday–Friday	353 (71.5%)	217 (73.8%)	232 (73.2%)	802 (72.6%)
Monday–Saturday	90 (18.2%)	45 (15.3%)	61 (19.2%)	196 (17.7%)
Monday–Sunday	51 (10.3%)	32 (10.9%)	24 (7.6%)	107 (9.7%)
Change in days per week facility is open since March 2011				
Increase	137 (27.7%)	96 (32.7%)	119 (37.5%)	352 (31.9%)
Decrease	31 (6.3%)	28 (9.5%)	26 (8.2%)	85 (7.7%)
No change	326 (66.0%)	170 (57.8%)	172 (54.3%)	668 (60.5%)
Providers per HIV-clinic day, median(Q1–Q3)				
Doctors	1 (1–2)	1 (1–2)	1 (1–2)	1 (1–2)
Nurses	4 (2–6)	5 (2–6)	6 (4–8)	5 (2–6)
Counselors	0 (0–1)	0 (0–1)	0 (0–1)	0 (0–1)
Lay Counselors	0 (0–1)	0 (0–1)	0 (0–1)	0 (0–1)
Expert Clients	2 (2–3)	2 (2–3)	2 (1–3)	2 (2–3)
All cadres combined	8 (6–13)	9 (6–13)	10 (6–13)	9 (6–13)
ART initiated at referral facility^a^	487 (98.6%)	289 (98.3%)	314 (99.1%)	1090 (98.6%)
ART refills provided at referral facility	494 (100%)	294 (100%)	317 (100%)	1105 (100%)
Providers who initiate ART				
Doctor only	57 (11.5%)	29 (9.9%)	25 (7.9%)	111 (10.0%)
Nurse only	153 (31.0%)	97 (33.0%)	94 (29.7%)	344 (31.1%)
Doctor and Nurse	277 (56.1%)	163 (55.4%)	195 (61.5%)	635 (57.5%)
N/A	7 (1.4%)	5 (1.7%)	3 (0.9%)	15 (1.4%)
Phone available to implement Linkage SOP^b^	465 (94.1%)	279 (94.9%)	312 (98.4%)	1056 (95.6%)
Monthly credit available to implement Linkage SOP, median (Q1–Q3)	SZL 150 (150–200)	SZL 150 (150–200)	SZL 150 (150–300)	SZL 150 (150–200)
Staff responsible for calling defaulters^c^				
Doctors	0 (0.0%)	0 (0.0%)	0 (0.0%)	0 (0.0%)
Nurses	239 (48.4%)	141 (48.0%)	200 (63.1%)	580 (52.5%)
Counselors	8 (1.6%)	12 (4.1%)	13 (4.1%)	33 (3.0%)
Lay Counselor/EC	321 (65.0%)	194 (66.0%)	188 (59.3%)	703 (63.6%)

^a^At the time of this study, Swaziland national treatment guidelines recommended ART initiation at CD4 < 350 cells/μl.

^b^Patient linkage, retention, and follow-up in HIV care standard operating procedures, Swaziland National AIDS Programme, 2012.

^c^More than one cadre could be responsible for calling clients who defaulted from their first or subsequent appointment to the HIV facility, in accordance with the Linkage SOP.

### Interview Eligibility and Participation

**Fig 3:**
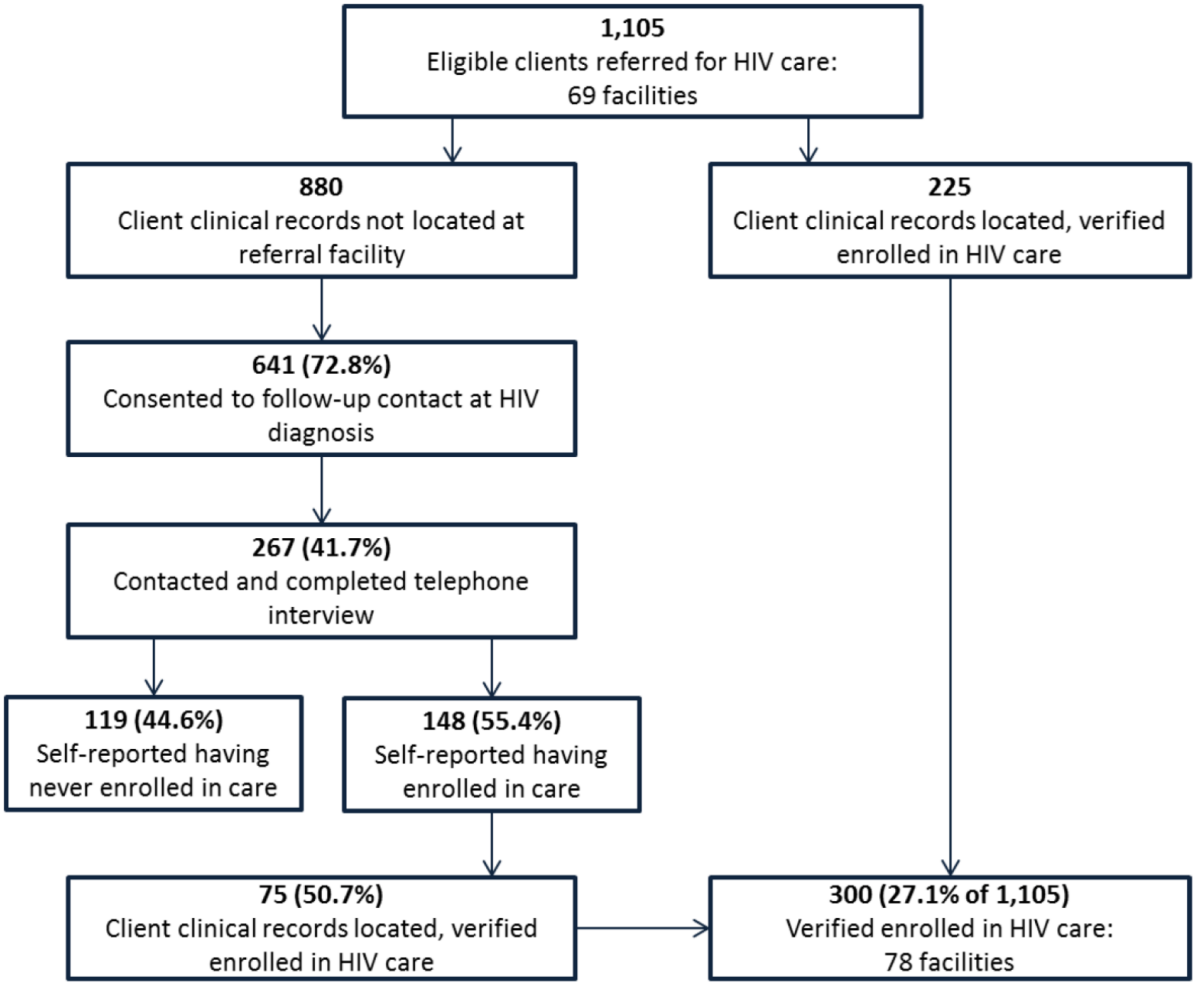
Eligible newly HIV diagnosed clients verified to have enrolled in HIV care.

Of the 1,105 clients, 225 were initially verified to have enrolled in HIV care at the facility to which they were referred. Of the 880 clients not initially verified to have enrolled at their referral facility, 641 (72.8%) had provided a telephone number and consented to follow-up contact at HIV diagnosis (Fig 3). Of these interview-eligible clients, 322 (50.2%) were contacted, of whom 267 (82.9%) consented to and completed a telephone interview. Telephone interviews were conducted a median (Q1–Q3) 957 (914–992) days from the date of diagnosis. Among the 880 clients not initially verified to have enrolled in care, interview rates (30.3%, 267/880) varied by age group among SHIMS females (age <25 years vs. ≥25 years, 18.6% vs. 33.3%; χ^2^ = 10.2; *P* = 0.001), but not among SHIMS and SOKA males (age <25 years vs. ≥25 years, 36.6% vs. 32.0%; χ^2^ = 0.64; *P* = 0.42); interview rates also did not vary by region (range 28.2%-31.9%; χ^2^ = 1.0; *P* = 0.80).

### Self-reported and Verified Enrollment in Care

Of 267 interviewed clients, 148 (55.4%) reported enrolling in HIV care, of whom 75 (28.1% of 267) were verified as having enrolled; 13 (4.9%) were verified at the referral facility and 62 (23.2%) were verified at an alternate facility (Table 2). Of 267 interviewed clients aged <25, 25–35, and >35 years of age, 15.0% (9/60), 28.2% (40/142), and 40.0% (26/65) were verified to have enrolled in HIV care, respectively (χ^2^ = 9.65; *P* = 0.008); verified enrollment in HIV care did not vary by study-gender group (χ^2^ = 1.01; *P* = 0.60) or region (χ^2^ = 0.91; *P* = 0.82).

**Table 2:** Interviewed client self-reported and verified enrollment in HIV care, and reasons for enrolling at an alternate facility or not enrolling in HIV care at all, by study-gender group.

Enrollment in HIV care	SHIMS Female (n = 105)	SHIMS Male (n = 71)	SOKA Male (n = 91)	All Clients Interviewed(n = 267)
Self-reported ever enrolled in HIV care				
Referral facility	26 (24.8%)	11 (15.5%)	14 (15.4%)	51 (19.1%)
Alternate facility	37 (35.2%)	24 (33.8%)	36 (39.6%)	97 (36.3%)
Any facility	63 (60.0%)	35 (49.3%)	50 (54.9%)	148 (55.4%)
Verified enrollment in HIV care^a^				
Referral facility	7 (6.7%)	2 (2.8%)	4 (4.4%)	13 (4.9%)
Alternate facility	21 (20.0%)	16 (22.5%)	25 (27.5%)	62 (23.2%)
Any facility	28 (26.7%)	18 (25.4%)	29 (31.9%)	75 (28.1%)
***Clients who enrolled at alternate facility***	***(n = 35)***	***(n = 23)***	***(n = 36)***	***(n = 94)***
Reasons for enrollment at alternate facility^b^				
Lives closer or transportation costs less	24 (68.6%)	19 (82.6%)	18 (50.0%)	61 (64.9%)
Receives better care or trusts providers more	14 (40.0%)	12 (52.2%)	14 (38.9%)	40 (42.6%)
Health care staff are more respectful	6 (17.1%)	6 (26.1%)	9 (25.0%)	21 (22.3%)
Shorter wait time or weekend services	4 (11.4%)	2 (8.7%)	8 (22.2%)	14 (14.9%)
Greater anonymity^c^	2 (5.7%)	1 (4.3%)	3 (8.3%)	6 (6.4%)
More familiar with facility or staff	3 (8.6%)	0 (0.0%)	1 (2.8%)	4 (4.3%)
Other reason	1 (2.9%)	0 (0.0%)	3 (8.3%)	4 (4.3%)
***Clients who had not enrolled in HIV care***	***(n = 42)***	***(n = 36)***	***(n = 38)***	***(n = 116)***
Reasons for not enrolling in HIV care^b^				
Feeling well, no need to go to HIV clinic	21 (50.0%)	15 (41.7%)	23 (60.5%)	59 (50.9%)
Too busy, no time	18 (42.9%)	14 (38.9%)	11 (28.9%)	43 (37.1%)
Has to wait too long to see a provider	5 (11.9%)	7 (19.4%)	9 (23.7%)	21 (18.1%)
Facility too far away / travel too expensive	4 (9.5%)	8 (22.2%)	4 (10.5%)	16 (13.8%)
Staff at health facilities are disrespectful	4 (9.5%)	2 (5.6%)	3 (7.9%)	9 (7.8%)
Negative opinion of ART^d^	1 (2.4%)	3 (8.3%)	1 (2.6%)	5 (4.3%)
Poor care quality or does not trust providers	3 (7.1%)	0 (0.0%)	1 (2.6%)	4 (3.4%)
Wants to remain anonymous^e^	3 (7.1%)	0 (0.0%)	2 (5.3%)	5 (4.3%)
Did not receive their HIV+ test results	3 (7.1%)	1 (2.8%)	1 (2.6%)	5 (4.3%)
Retested HIV negative	2 (4.8%)	2 (5.6%)	1 (2.6%)	5 (4.3%)
No one followed up after diagnosis	0 (0.0%)	1 (2.8%)	4 (10.5%)	5 (4.3%)
Other reason	1 (2.4%)	2 (5.6%)	3 (7.9%)	6 (5.2%)

^a^Abstraction teams returned to referral facility or visited alternate facility and verified enrollment in care.

^b^Clients who reported one or more reasons; response options were not read to client; more than one reason could be given.

^c^Greater confidentiality; does not know people at the facility; lives further away from the facility.

^d^Does not believe HIV treatment is effective; believes HIV treatment has severe side effects; fears ART, being treated by a traditional healer.

^e^Does not want others to know HIV status; not yet disclosed status to partner; fears separation from spouse or partner.

### Reasons for Enrolling at an Alternate Facility or not Enrolling in Care

Of 94 interviewed clients who reported reasons for enrolling at an alternate facility, the most common reasons included greater convenience, lower costs, and perceived higher quality of care and respect by staff (Table 2). Of 116 interviewed clients who provided reasons for not enrolling in HIV care, the most common reasons included perceived good health, lack of time, inconvenience, and transportation costs (Table 2). Few clients reported never enrolling in HIV care because of negative perceptions of treatment or the quality of HIV care. Of the 119 interviewed clients who reported having never enrolled in HIV care, 87 (73.1%) also reported wanting to enroll in care and 78 (65.5%) accepted a referral and appointment.

### Verified Enrollment in HIV Care

Study personnel visited 92 facilities to verify enrollment, 69 referral and 23 alternate. At least one client was verified to have enrolled in each of 78 facilities: 61 (88.4%) referral and 17 (73.9%) alternate. Of 1,105 clients, study personnel verified that 300 (27.1%) enrolled in HIV care (Fig 3); 136 (27.5%) SHIMS females, 73 (24.8%) SHIMS males, and 91 (28.7%) SOKA males. Of the 300 enrolled clients, 84 (28.0%) enrolled at the 17 alternate facilities, white referral forms were located for 53 (17.7%), and medical charts were located and used for data abstraction on 266 (88.7%). Of the remaining 34 clients, pre-ART or ART registers (n = 21) and electronic medical records (n = 13) were used for data abstraction.

### Client Correlates

**Fig 4:**
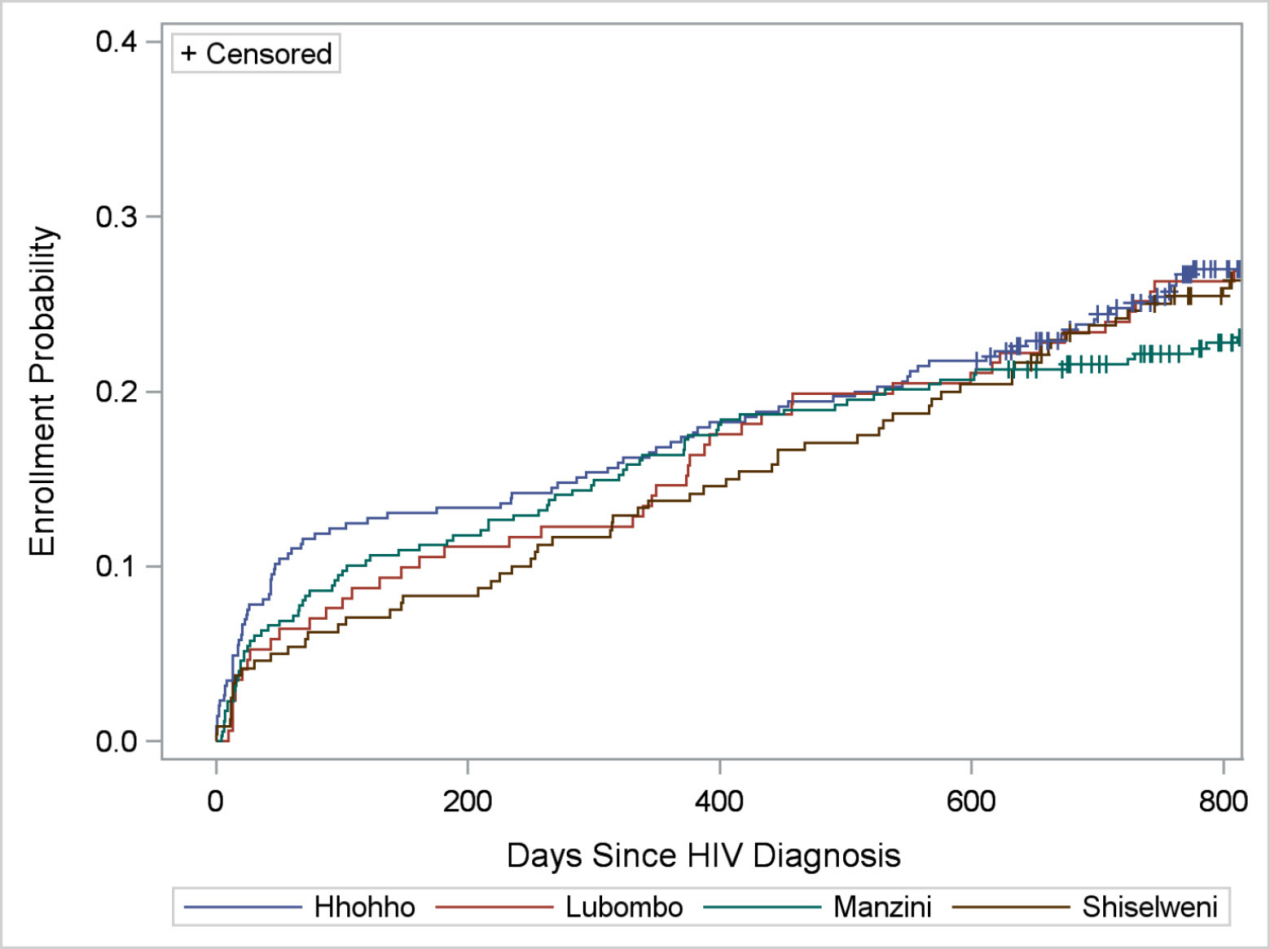
Cumulative verified enrollment in HIV care, by region of facility. Log-rank test for differences in functions = 0.73; *P* = 0.87.

**Fig 5:**
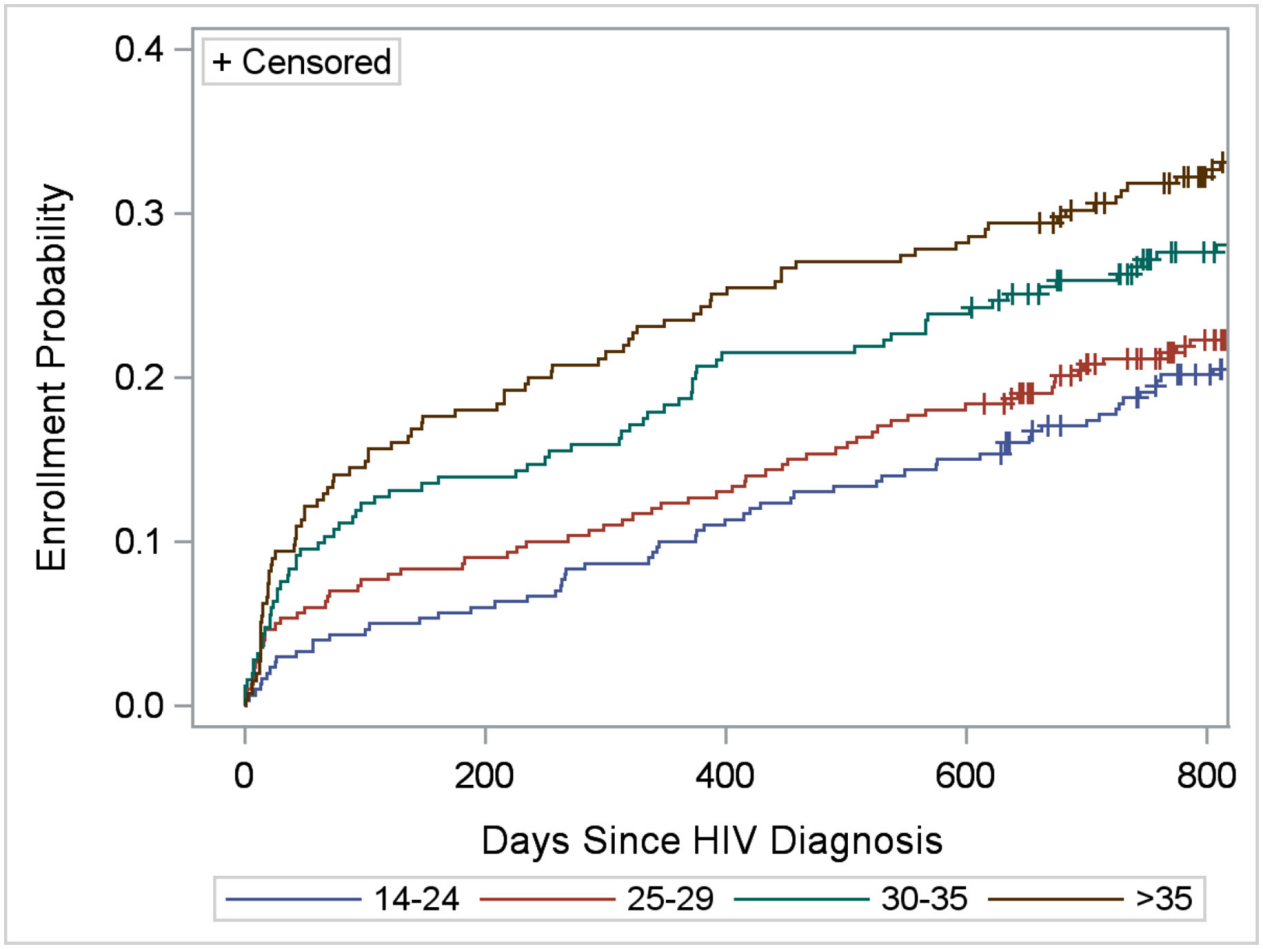
Cumulative verified enrollment in HIV care, by age group (years). Log-rank test for differences in functions = 18.39; *P* = 0.0004.

**Fig 6:**
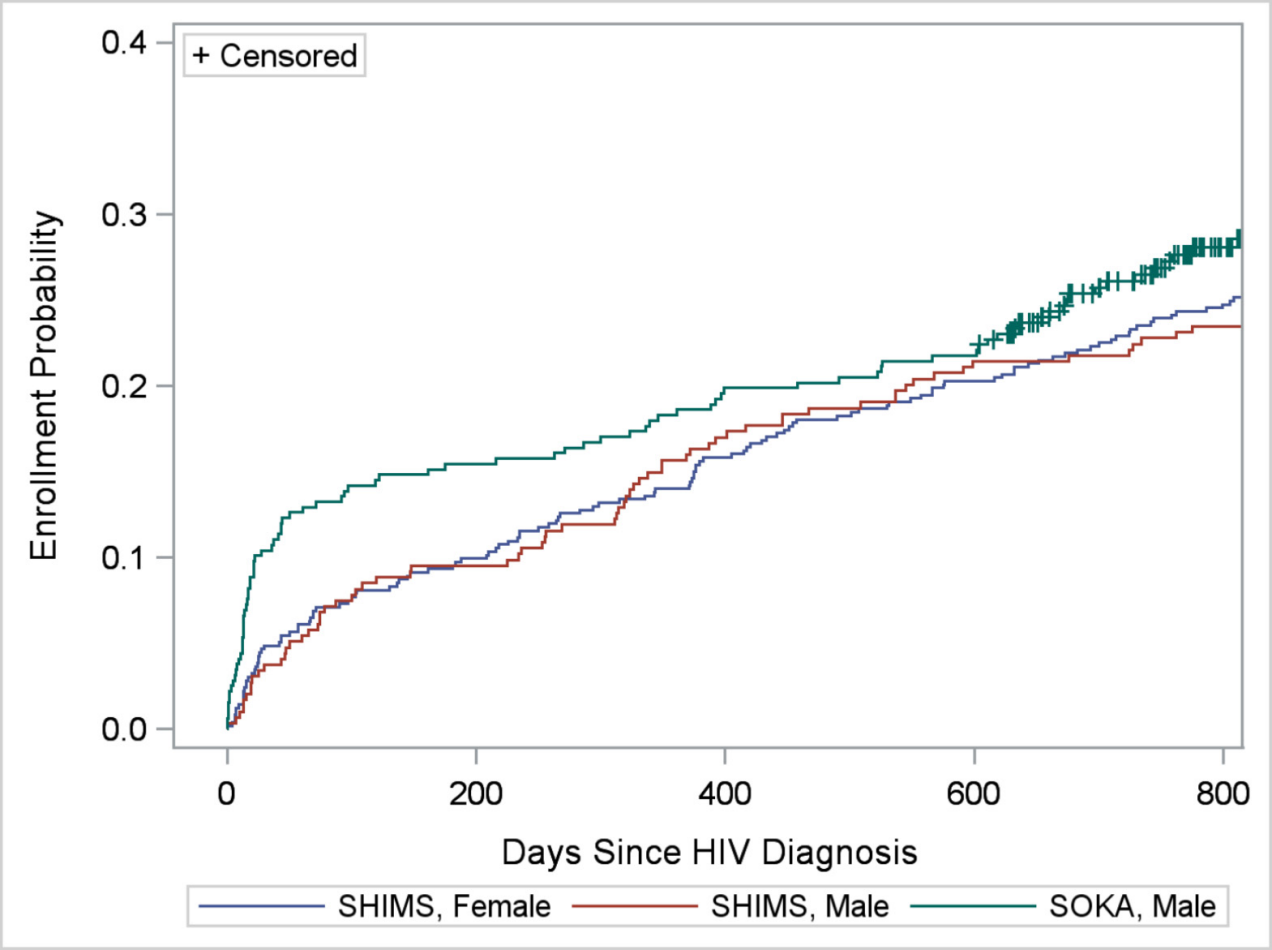
Cumulative verified enrollment in HIV care, by study-gender group. See text for evaluation of differences in functions.

Enrollment in HIV care within 3, 6, 12, 18, and 24 months of diagnosis was verified for 100 (9.0%), 124 (11.2%), 174 (15.7%), 221 (20.0%) and 265 (24.1%, including censored observations) clients, respectively. Kaplan-Meier survival (enrollment-in-care) functions did not vary by region (*LR* = 0.73; *P* = 0.87), but varied by age-group (*LR* = 18.39; *P* = 0.0004) (Figs 4 and 5). Clients >35 years of age were more likely to enroll in care compared with those 14–24 (*LR* = 14.74; *P* = 0.0001) and 25–29 (*LR* = 10.60; *P* = 0.001) years of age. Kaplan-Meier functions indicating greater enrollment probability among older age groups remained statistically significant when controlling for region, urban/rural location of facility, and study-gender group (data not shown). Taking censoring into consideration, within two years of diagnosis, 18.8%, 21.2%, 26.3%, and 31.4% of clients 14–24, 25–29, 30–35, and >35 years of age, respectively, were verified to have enrolled in care. Differences in enrollment by study-gender group depended on time since diagnosis (Fig 6). Proportionally more SOKA than SHIMS clients enrolled in care 90 and 182 days after diagnosis (90 days: 13.2% vs. 7.4%, χ^2^ = 9.48, *P* = 0.002; 182 days: 15.5% vs. 9.5%, χ^2^ = 7.96; *P* = 0.005); at 600 days after diagnosis prior to the first censored observation, no differences were observed between SOKA and SHIMS clients (χ^2^ = 0.15; 21.8% vs. 20.7%; *P* = 0.70) (Fig 6).

### Facility Correlates

**Fig 7:**
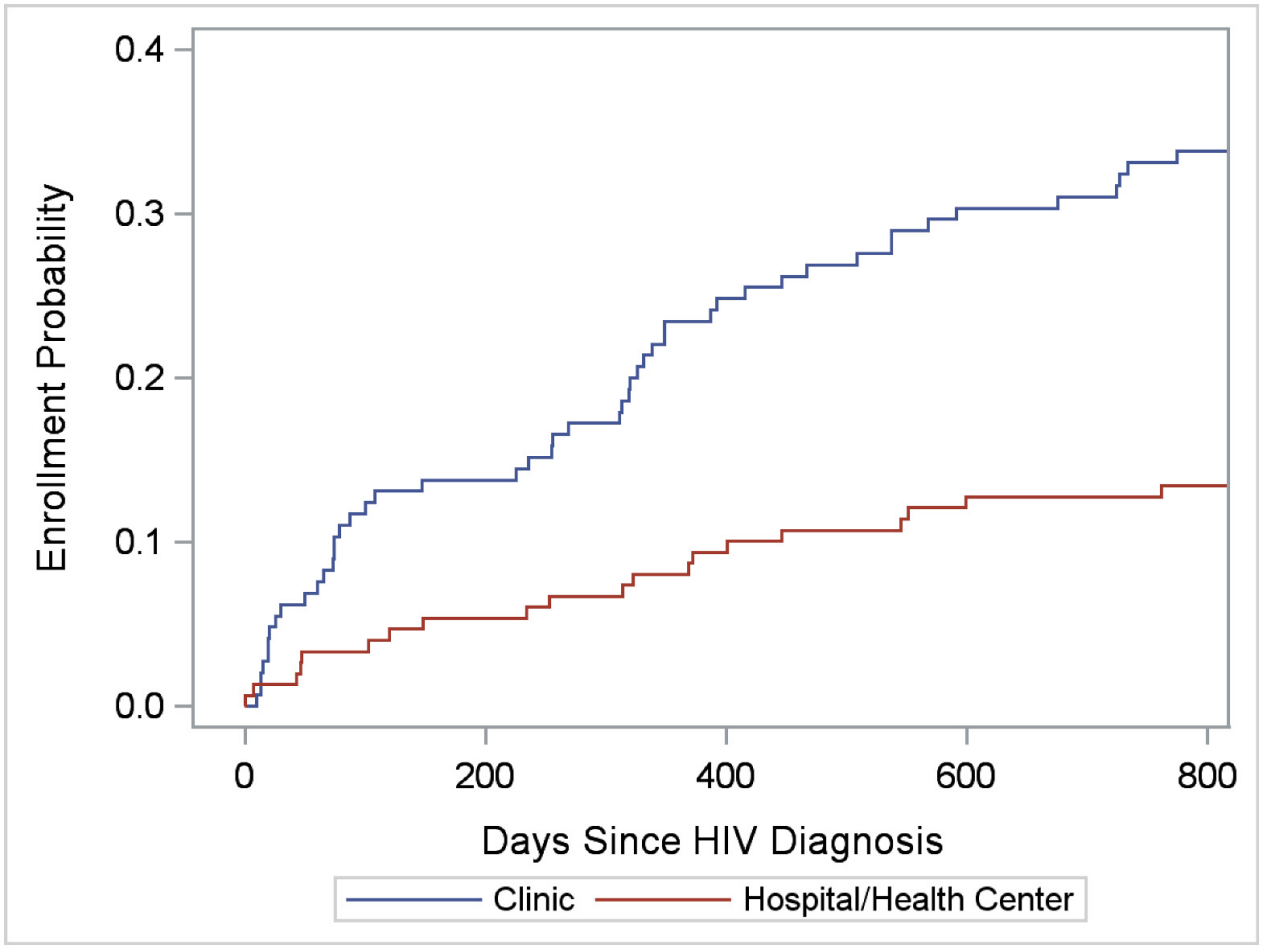
Cumulative verified enrollment in HIV care, by type of health facility, SHIMS male clients only. Log-rank test for differences in functions = 18.47; *P* < 0.0001.

**Fig 8:**
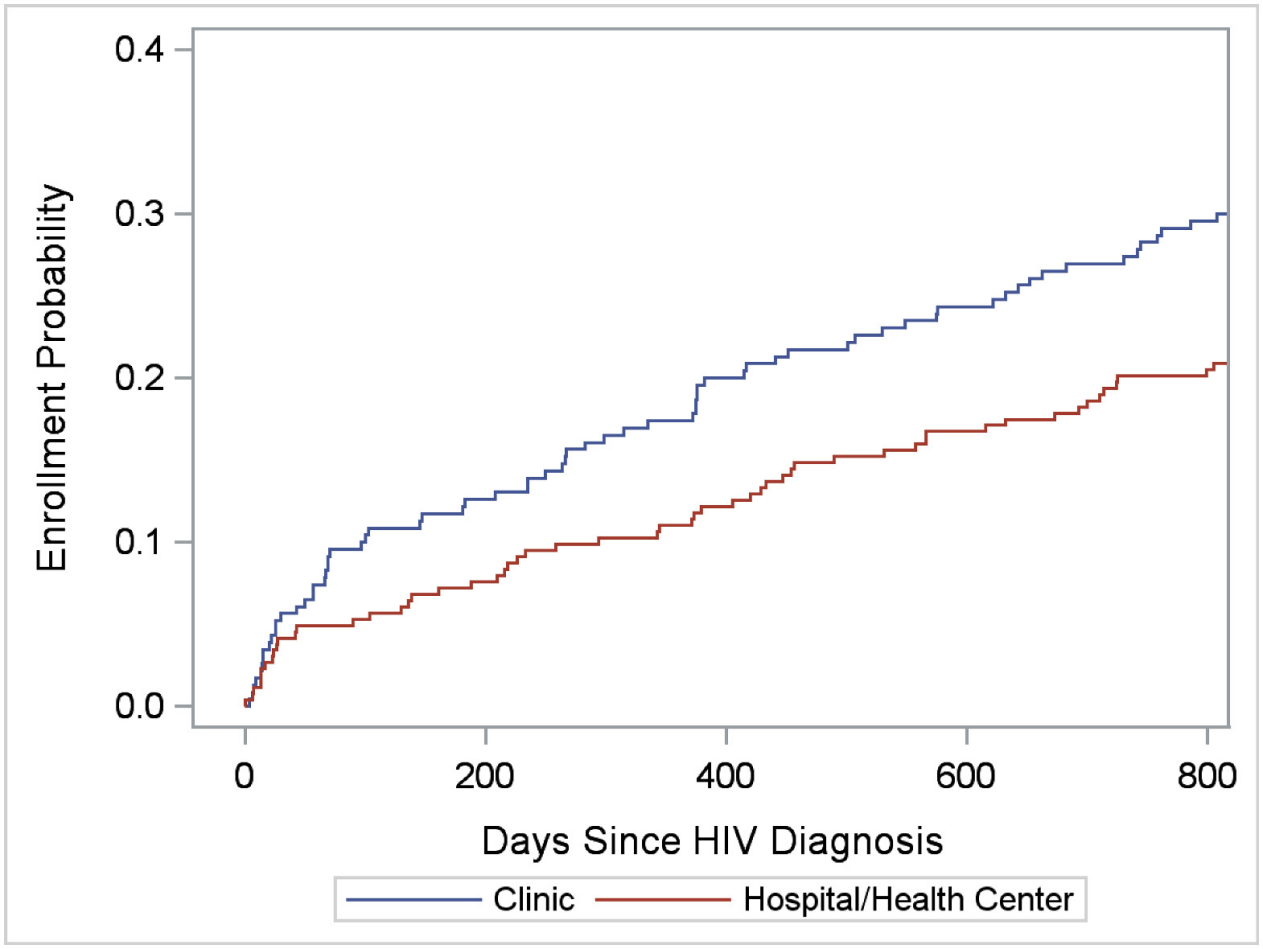
Cumulative verified enrollment in HIV care, by type of health facility, SHIMS female clients only. Log-rank test for differences in functions = 6.09; *P* = 0.014.

**Fig 9:**
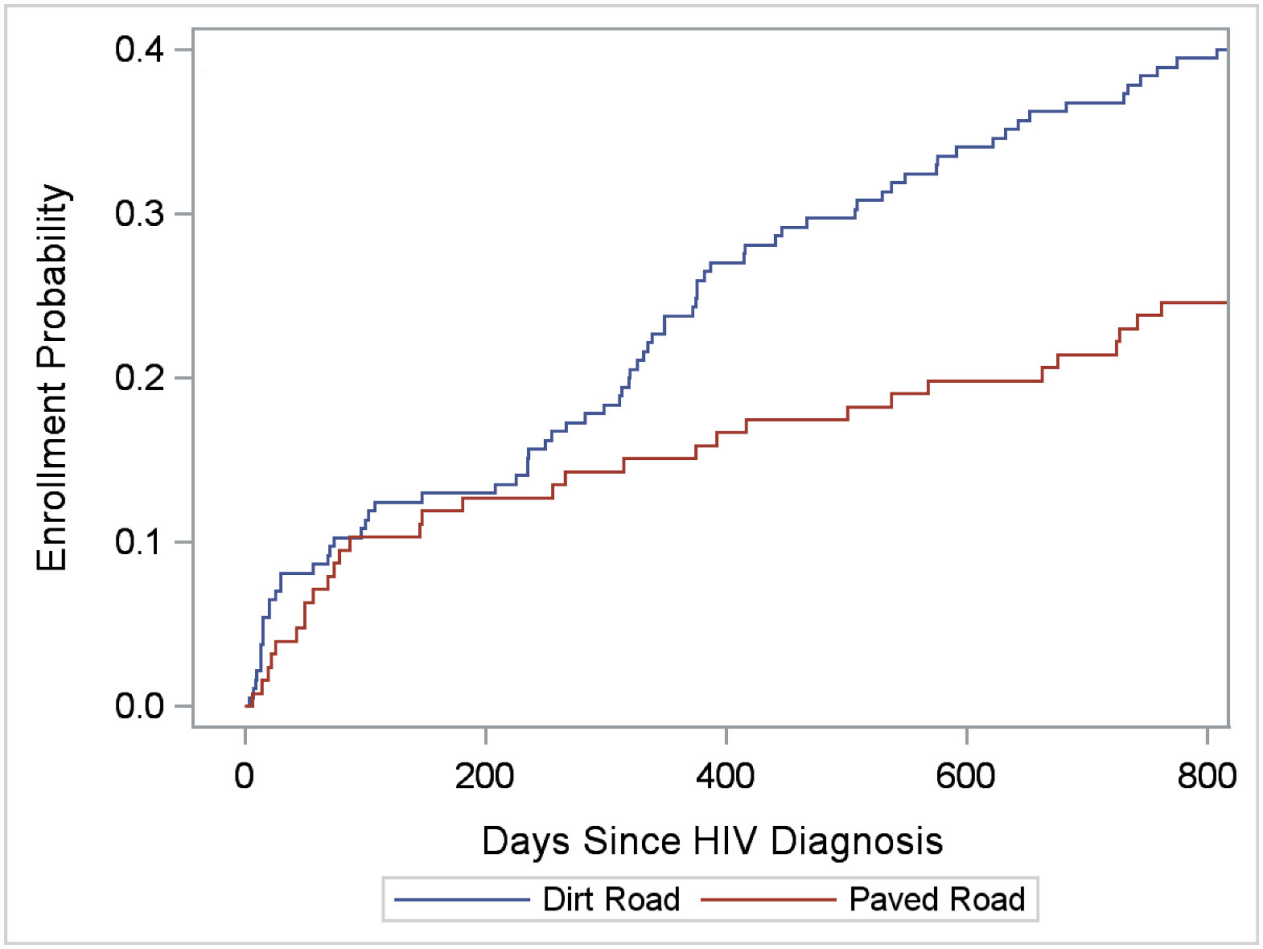
Cumulative verified enrollment in HIV clinics located in rural and peri-urban areas, by road condition serving the clinic, SHIMS clients only. Log-rank test for differences in functions = 8.01; *P* = 0.005.

To evaluate facility correlates of enrollment in care, for enrolled clients, we selected the enrollment facility (referral or alternate) to analyze; for non-enrolled clients, we selected the referral facility. Thus, 86 facilities were available for individual-level analyses, including 78 facilities with at least one enrollment and 8 referral facilities without any enrollments. The 86 facilities were equally distributed in the four regions (range: 22.1%-27.9%), and included 72 clinics, 8 hospitals, 5 health centers, and 1 public health unit. Kaplan-Meier functions indicated greater enrollment probability in clinics than hospitals and health centers among SHIMS males (*LR* = 18.47; *P* < 0.0001) and females (*LR* = 6.09; *P* = 0.014) (Figs 7 and 8), but not among SOKA males (*LR* = 0.45; *P* = 0.50) (data not shown). Among SHIMS males, enrollment in hospitals and health centers was particularly low (Fig 7). Kaplan-Meier functions indicating greater clinic-enrollment probability among SHIMS clients remained statistically significant when controlling for age-group, region, urban/rural facility location, and after restricting the analysis to referral facilities alone (data not shown). Of clinics located in rural and peri-urban areas only (clinics on paved roads, n = 20; clinics on dirt roads, n = 34), enrollment in care among SHIMS clients (n = 311) was greater in those clinics located on dirt roads (*LR* = 8.01; *P* = 0.005) (Fig 9) (analyses were restricted to SHIMS clients because few SOKA clients were referred to or enrolled in clinics served by dirt roads). Kaplan-Meier functions indicating greater enrollment probability in clinics on dirt roads remained statistically significant after controlling for age-group, gender, region, and restricting the analysis to referral facilities alone (data not shown).

### Estimated Enrollment-in-care Adjusted for Lost to Follow-up and Non-response

Of the 613 clients who were not initially verified to have enrolled in care and not interviewed (<25 years, n = 183; 25–35 years, n = 305; >35 years, n = 125), 164 are estimated to have enrolled in HIV care by applying age-group-specific verified enrollment probabilities of interviewed clients (<25 years, 0.150; 25–35 years, 0.282; >35 years, 0.400). Assuming the cumulative enrollment distribution of the 300 enrolled-verified clients apply to these 164 additional clients, the age-group-adjusted number (%) of SHIMS and SOKA clients estimated to have enrolled in care at any facility in Swaziland is 464 (42.0%) overall, and within 3, 6, 12, 18, and 24 months of diagnosis, 155 (14.0%), 192 (17.4%), 269 (24.3%), 342 (30.9%), and 409 (37.0%), respectively.

### Characteristics at Enrollment

The 300 clients enrolled in HIV care at a median (Q1–Q3) age of 32 years (26–38) and 285 days (43–566) after HIV diagnosis. Of clients with documented baseline CD4 count (n = 292, 97.3%) or WHO clinical stage (n = 279, 93.0%), the median (Q1–Q3) CD4 count at enrollment was 280 (165–420); 16.1% were classified with AIDS (WHO stage III or IV); and 66.0% were ART eligible (WHO stage III/IV or CD4 ≤ 350 cells/μl). Of enrolled clients, 208 (69.3%) were initiated on ART.

### Compliance with the Linkage SOP

Of the 1,105 clients referred to 69 facilities, pink HTC forms were located on 517 (46.8%); 106 (9.6%) had their expected arrival date recorded in the appointment register; and 3 (0.3%) and 54 (4.9%) were called before or within two weeks after their enrollment appointment (clinic staff documented speaking with 32 of the 54 called). Of the 517 HTC forms, 351 (67.9%) included telephone numbers and consent for follow-up contact; of these, 333 (94.9%) were of clients who did not enroll in care within 21 days of diagnosis and who should have received a second follow-up call. Of these 333 appointment defaulters, 27 (8.1%) were called within two weeks of their missed appointment. Of the 267 interviewed clients, 251 (94.0%) recalled being referred to HIV care at the time of their diagnosis, of whom 181 (72.1%) accurately reported the name of the referral facility on the HTC form. Few interviewed clients, however, reported receiving linkages services from the referral facility (Table 3). Of the 32 documented clients who were successfully contacted by clinic staff after their enrollment appointment, 16 (50%) were verified to have enrolled in HIV care.

**Table 3:** Interviewed client self-reported linkage services, by study-gender group^a^.

Linkage Services Received	SHIMS Female (n = 105)	SHIMS Male (n = 71)	SOKA Male (n = 91)	All Clients Interviewed (n = 267)
Reported being referred to HIV care at diagnosis.	102 (97.1%)	65 (91.5%)	84 (92.3%)	251 (94.0%)
Accurately recalled the same referral facility noted on the HTC form.^b^	74 (70.5%)	47 (66.2%)	60 (65.9%)	181 (67.8%)
Received an SMS text reminder to enroll in HIV care.	9 (8.6%)	5 (7.0%)	7 (7.7%)	21 (7.9%)
Received a phone call from the referral facility to enroll in HIV care.	8 (7.6%)	4 (5.6%)	12 (13.2%)	24 (9.0%)
Received a home visit from a referral facility representative.	10 (9.5%)	3 (4.2%)	0 (0.0%)	13 (4.9%)
Spoke at home with referral facility representative about enrolling in care.	6 (5.7%)	3 (4.2%)	0 (0.0%)	9 (3.4%)

^a^Interviews were conducted a median (Q1–Q3) of 957 (914–992) days from diagnosis.

^b^Of the 251 clients who recalled being referred to care and treatment at the time of their diagnosis, 181 (72.1%) accurately reported the name of the facility where they had been referred.

### Visitation without Enrollment in HIV Care

Of the 1,105 clients, 49 (4.4%) had visited an HIV-care facility but were not defined as enrolled because (1) they were not clinically staged and did not have a CD4 test (n = 37), or (2) they had a CD4 test (without WHO clinical staging) but did not return to the clinic to receive their result (n = 12). Of these 49 visitors (19 SHIMS, 30 SOKA), the only documentation found for nine was the white referral form. The 40 clients who were documented either on a register or medical chart visited the facility a median (Q1–Q3) of 1 day (0–43) after their diagnosis. Of the 49 clients who visited but did not enroll at the facility, 37 (75.5%) provided a telephone number and consented to be contacted, of whom two had been called.

### Data Source Availability

Of the 92 facilities visited, the following data sources were available to abstraction teams to identify enrolled clients (% of facilities visited): medical chart chronic-care files (92.4%); expert clients who helped staff identify clients and locate records (78.3%); stored HTC referral forms (76.1%); electronic medical records (30.4%). In addition, of the expected 108 months of searchable appointment, pre-ART, and ART registers for years 2011–2013 at each facility (108 = 3 registers X 12 months X 3 years); the median (Q1–Q3) number of months of searched registers was 92 (72–103) (some registers were missing pages for ≥1 month and sometimes ≥1 registers for a given year could not be found). Data-source availability was considered inadequate for one of the 92 facilities because none of the following data sources were available: expert clients, electronic medical record system, and appointment, pre-ART, and ART registers for all years 2011–2013.

## Discussion

### Enrollment in HIV Care

We found that of over one thousand clients newly HIV diagnosed in Swaziland in 2011 and 2012, less than one in four (24.1%) were verified to have enrolled in care within two years of their diagnosis. Even after adjusting for lost to follow-up and non-response, few clients (37.0%) were estimated to have enrolled in HIV care within two years of their diagnosis. Similarly low rates of verified enrollment in HIV care were observed for both men and women diagnosed in their homes, among men seeking VMMC services, in each of the four regions of Swaziland, and in all age groups. Although older persons in our sample were more likely to have enrolled in HIV care, less than one in three (31.4%) in the oldest age group (>35 years) were verified as having enrolled in care within two years of diagnosis. Our findings on low enrollment in HIV care following HIV diagnosis is similar to findings from other studies in Sub-Saharan Africa (range: 10%-51%), including one study in the Shiselweni region of Swaziland, conducted of persons tested in community settings who were not provided follow-up linkage services [16,21–28,30]. Enrollment-in-care rates of persons tested in community settings are notably higher (range: 68%-100%) from studies conducted in communities located near health facilities or whose residents received routine outreach, linkage, or mobile health services [13–15,30].

Of the few SOKA and SHIMS clients who were verified as having enrolled in HIV care, two-thirds were already eligible for ART (at CD4 < 350 cells/μl). Consistent with the Swaziland National ART Program Evaluation, our findings suggest that many newly HIV-diagnosed Swazis delay their enrollment in care for years, and as a result, are initiated on ART late in the course of their HIV disease [18]. Thus, considerable HIV-related morbidity and mortality, and HIV transmission to sex partners and offspring might be averted by reducing this delay [1–5]. National procedures intended to increase early enrollment in care among SOKA and SHIMS clients either were not sufficiently implemented and documented, or if applied in accordance with the SOP, did not apparently work for most clients. The failure in either application or efficacy of these linkage procedures was particularly acute for clients under 25 years of age, less than one in five (18.8%) of whom were verified to have enrolled in care within two years of their diagnosis.

### Missed Opportunities to Provide Linkage Services

We found that there was considerable opportunity to intervene and help clients enroll early in care: nearly three in four (71.4%) of the 1,105 clients provided a telephone number and consented to follow-up contact at the time of their diagnosis. Our staff were able to call and interview many of these clients >900 days after providing their telephone number. Of those contacted, nearly nine in ten agreed to discuss with persons whom they didn’t know whether they had enrolled in HIV care and reasons for not enrolling in care. Of clients who reported never enrolling in HIV care, two-thirds reported wanting to enroll and accepted a new appointment and referral to an HIV-care facility. Our findings suggest that had the calls been conducted soon after diagnosis, linkage services could have been provided to most clients [19].

### Delivery and Storage of HTC Referral Forms

In accordance with the Linkage SOP, the referral form delivery system is essential to providing linkage services because it is the primary means for facility staff to make appointments for clients, to initiate reminder and missed-appointment calls, and to prompt home visits [19]. We found pink HTC forms for only half of the clients at facilities to which they were referred. Although the percentage of forms that were delivered and available for use at the time of the enrollment appointment is unknown, discussion with facility staff suggest that many forms were not delivered. We also learned at several facilities that referral forms often arrived after clients‘ expected arrival dates, and thus could not be used to remind clients of their appointment. Of clients who enrolled in HIV care, we found white copies of their HTC forms on less than one in five. Our findings, thus, underscore considerable challenges and limitations of using referral forms as a means to prompt linkage services by staff at HIV-care facilities as well as to document enrollment in care.

### Reminder and Appointment Defaulter Calls

Despite the availability of many HTC forms that facility staff could have used to call clients to remind them of their appointment or missed appointment, documentation of these calls were found for very few clients. Notably, nearly all clients were referred to facilities that had nurses and counselors trained to implement the Linkage SOP, and cell phones and adequate airtime credit to call referred clients. Although we cannot rule out that undocumented calls or texts were made in accordance with the Linkage SOP, we believe that most clients were not called because very few were located in the appointment register (the means used to prompt calls), and because very few interviewed clients who missed their appointment reported receiving a telephone call or text reminder from the facility.

### Differences in Early Enrollment in HIV Care

Interestingly, proportionally more SOKA than SHIMS clients enrolled in HIV care within 3–6 months of diagnosis, but cumulative enrollment-in-care between the study groups converged overtime. Observed differences in early enrollment in care might be attributed to (1) SOKA sites that were often co-located at HIV-care facilities, making early enrollment more convenient at these facilities; (2) young men seeking circumcision services may initially have had a greater propensity to seek HIV care than their general-population peers who accepted, but had not sought out HTC services they received at home; and (3) some SOKA clients receiving linkage services from counselors, including escort services at co-located facilities.

### Decentralized HIV Care

The well-established importance of task shifting and decentralized HIV care is supported in our findings of substantially greater enrollment at clinics than hospitals and health centers to which SHIMS clients were referred; and in rural and peri-urban areas, at clinics served by dirt roads than clinics served by paved roads (in Swaziland, all highways and a large majority of urban roads are paved) [1]. These associations were not observed for SOKA clients who were more likely than SHIMS clients to be referred to urban facilities served by paved roads and often co-located at circumcision sites. For SHIMS clients diagnosed at their homes, these findings were not unexpected given substantially greater congestion and longer wait times observed at hospitals that are mostly served by paved roads at plausibly greater distances from client residences. Our findings suggest that decentralized HIV care at presumably more convenient clinics may be particularly important for early enrollment in care among men diagnosed at home and other community settings.

### Enrolling in Alternate Facilities

We found that over one quarter (28%) of clients who were verified to have enrolled in HIV care enrolled at facilities to which they were *not* referred. Of interviewed clients who enrolled at these alternate facilities, most reported that they choose the facility for reasons of convenience; however, many also perceived that care was better at that facility and that staff were more respectful to clients [27,31,32]. These findings underscore the importance for counselors to explore perceived barriers to care at nearby facilities and ensure that concerns about the quality of care and the treatment of clients are explored and addressed. Our findings also suggest that studies that do not consider enrollment at non-referral facilities may substantially underestimate enrollment in care [6,7].

### Visitation without Enrollment in HIV Care

We found that some clients had visited HIV-care facilities and did not meet our definition of enrollment in care. This was particularly true of SOKA clients, of whom nearly one in ten had visited the facility on or shortly after the day of their diagnosis. The relatively high rate of facility visitation without receipt of clinically meaningful services is discouraging because these clients were “linked” to care. Information on the individual circumstances of these clients and the context of their facility visits was not available; however, facility-level factors (e.g., long wait times, limited hours of operation, etc.) may have contributed to non-enrollment [1,27,31]. Most (75%) of these clients provided a telephone number and consented to be contacted; if they were called in accordance the Linkage SOP, it is possible that clinic staff might have helped identify and resolve barriers to care at that facility, or facilitated enrollment at a more suitable facility.

### Limitations

Our findings are subject to three important limitations. First, our enrollment estimate adjusted for lost to follow-up and non-response should be considered a minimum estimate because of incomplete data sources at some facilities and because of human error in identifying enrolled clients. However, nearly all facilities visited had adequate data sources with which to search for, match, and verify client enrollment. In addition, senior-investigator audits conducted at 28 facilities for 364 (41.4% of 880) clients who were not initially verified as having enrolled in care, identified only 13 (3.6%) additional clients who enrolled in care. Although we undoubtedly missed some enrolled clients, our extensive procedures and audits suggest the underestimate is small. Second, findings from this study are subject to omissions and errors in clinical records, correctly matching clients with facility data sources, and accurately transcribing clinical information onto study forms. Senior-investigator audits of 120 (40%) forms on enrolled clients at 15 facilities, however, found only one that was abstracted on a non-matched client; 8 (6.6%) had one or more transcription errors. Finally, our findings are restricted to the sample of SHIMS and SOKA clients diagnosed through home-based HTC and at circumcision sites in Swaziland in 2011 and 2012 on whom eligible HTC forms were located. Our findings may not adequately represent SHIMS and SOKA populations, other HTC populations diagnosed during this period, and linkage-service practices and enrollment in care outcomes of client cohorts diagnosed more recently.

### Recommendations

Low compliance with the Linkage SOP for SHIMS and SOKA clients in 2011 and 2012 might have been attributed to (1) a specimen transport system that was unable to routinely provide referral forms to facilities in a timely manner, and (2) overburdened clinical staff who through their training and orientation are unfamiliar with providing services to patients not yet under their care. For these reasons, expansion of linkage responsibilities in Swaziland should be considered for HTC providers who are in a better position to provide timely linkage services that meet the individual circumstances and needs of their clients. Currently recommended linkage services that HTC providers should consider adopting include transport, escort, treatment navigation, and case management [1,33,34]. Delivered by peer counselors who receive specialized training in HIV-care and psychosocial support, these services could be tailored to address the two most frequently reported barriers to enrolling in HIV care in our study and elsewhere: perceived good health and inconvenience [1,26,27,31–34]. These and other recommended linkage interventions are under evaluation in Swaziland as part of new community- (CommLink) and facility-based (Link4Health) programs. CommLink is implemented by peer counselors who provide escort, treatment-navigation, and linkage-case-management services to persons diagnosed in community settings. Link4Health is a cluster randomized trial of a combination of five interventions (e.g., point-of-care CD4 and accelerated ART initiation) to improve enrollment in care among patients diagnosed in health-care facilities [35].

## Conclusions

In the first study of its kind in Swaziland, we found near universal low compliance with national guidelines and procedures to improve early enrollment in HIV care. Of over one thousand newly HIV diagnosed clients, we estimate after adjusting for lost to follow-up and non-response that less than four in ten enrolled in HIV care within two years of their diagnosis. Of those who enrolled in HIV care, most enrolled late in the course of their HIV disease. To increase the potential of ART to reduce HIV transmission, and HIV-related morbidity and mortality, our findings are a call to action to substantially improve linkage services and early enrollment in HIV care in Swaziland.
