# Correction: Enrollment in HIV Care Two Years after HIV Diagnosis in the Kingdom of Swaziland: An Evaluation of a National Program of New Linkage Procedures

**DOI:** 10.1371/journal.pone.0152108

**Published:** 2016-03-17

**Authors:** The PLOS ONE Staff

The caption for Table 1, “Clisssent and referral facility characteristics, by study-gender group,” should be “Client and referral facility characteristics, by study-gender group.” The publisher apologizes for the error. Please see Table 1 with the correct caption here.

**Table 1:** Client and referral facility characteristics, by study-gender group.

Characteristic		SHIMS Female (N = 494)	SHIMS Male (N = 294)	SOKA Male (N = 317)	All Clients (N = 1105)
Age at diagnosis, median (Q1-Q3)		26 (22–33)	32 (27–38)	29 (25–35)	29 (24–35)
Age at diagnosis (years)					
	<25	204 (41.3%)	37 (12.6%)	58 (18.3%)	299 (27.1%)
	25–29	122 (24.7%)	72 (24.5%)	105 (33.1%)	299 (27.1%)
	30–35	81 (16.4%)	84 (28.6%)	87 (27.4%)	252 (22.8%)
	>35	87 (17.6%)	101 (34.4%)	67 (21.1%)	255 (23.1%)
Region of referral facility					
	Hhohho	148 (30.0%)	101 (34.4%)	102 (32.2%)	351 (31.8%)
	Lubombo	83 (16.8%)	51 (17.3%)	38 (12.0%)	172 (15.6%)
	Manzini	135 (27.3%)	81 (27.6%)	126 (39.7%)	342 (31.0%)
	Shiselweni	128 (25.9%)	61 (20.7%)	51 (16.1%)	240 (21.7%)
Type of referral facility					
	Government (non-military)	369 (74.7%)	231 (78.6%)	189 (59.6%)	789 (71.4%)
	Faith-based	91 (18.4%)	39 (13.3%)	23 (7.3%)	153 (13.8%)
	Private	24 (4.9%)	15 (5.1%)	46 (14.5%)	85 (7.7%)
	Non-governmental Organization	8 (1.6%)	4 (1.4%)	46 (14.5%)	58 (5.2%)
	Military	2 (0.4%)	5 (1.7%)	13 (4.1%)	20 (1.8%)
Class of referral facility					
	Hospital	166 (33.6%)	109 (37.1%)	105 (33.1%)	380 (34.4%)
	Health Center	92 (18.6%)	43 (14.6%)	36 (11.4%)	171 (15.5%)
	Clinic	227 (46.0%)	137 (46.6%)	160 (50.5%)	524 (47.4%)
	Public Health Unit	9 (1.8%)	5 (1.7%)	16 (5.0%)	30 (2.7%)
Location of referral facility					
	Urban	176 (35.6%)	110 (37.4%)	188 (59.3%)	474 (42.9%)
	Peri-urban	66 (13.4%)	56 (19.0%)	45 (14.2%)	167 (15.1%)
	Rural	252 (51.0%)	128 (43.5%)	84 (26.5%)	464 (42.0%)
Referral facility on a paved road		370 (74.9%)	217 (73.8%)	292 (92.1%)	879 (79.5%)
Days per week HIV services provided					
	Monday–Friday	353 (71.5%)	217 (73.8%)	232 (73.2%)	802 (72.6%)
	Monday–Saturday	90 (18.2%)	45 (15.3%)	61 (19.2%)	196 (17.7%)
	Monday–Sunday	51 (10.3%)	32 (10.9%)	24 (7.6%)	107 (9.7%)
Change in days per week facility is open since March 2011					
	Increase	137 (27.7%)	96 (32.7%)	119 (37.5%)	352 (31.9%)
	Decrease	31 (6.3%)	28 (9.5%)	26 (8.2%)	85 (7.7%)
	No change	326 (66.0%)	170 (57.8%)	172 (54.3%)	668 (60.5%)
Providers per HIV-clinic day, median(Q1–Q3)					
	Doctors	1 (1–2)	1 (1–2)	1 (1–2)	1 (1–2)
	Nurses	4 (2–6)	5 (2–6)	6 (4–8)	5 (2–6)
	Counselors	0 (0–1)	0 (0–1)	0 (0–1)	0 (0–1)
	Lay Counselors	0 (0–1)	0 (0–1)	0 (0–1)	0 (0–1)
	Expert Clients	2 (2–3)	2 (2–3)	2 (1–3)	2 (2–3)
	All cadres combined	8 (6–13)	9 (6–13)	10 (6–13)	9 (6–13)
ART initiated at referral facility^a^		487 (98.6%)	289 (98.3%)	314 (99.1%)	1090 (98.6%)
ART refills provided at referral facility		494 (100%)	294 (100%)	317 (100%)	1105 (100%)
Providers who initiate ART					
	Doctor only	57 (11.5%)	29 (9.9%)	25 (7.9%)	111 (10.0%)
	Nurse only	153 (31.0%)	97 (33.0%)	94 (29.7%)	344 (31.1%)
	Doctor and Nurse	277 (56.1%)	163 (55.4%)	195 (61.5%)	635 (57.5%)
	N/A	7 (1.4%)	5 (1.7%)	3 (0.9%)	15 (1.4%)
Phone available to implement Linkage SOP^b^		465 (94.1%)	279 (94.9%)	312 (98.4%)	1056 (95.6%)
Monthly credit available to implement Linkage SOP, median (Q1-Q3)		SZL 150 (150–200)	SZL 150 (150–200)	SZL 150 (150–300)	SZL 150 (150–200)
Staff responsible for calling defaulters^c^					
	Doctors	0 (0.0%)	0 (0.0%)	0 (0.0%)	0 (0.0%)
	Nurses	239 (48.4%)	141 (48.0%)	200 (63.1%)	580 (52.5%)
	Counselors	8 (1.6%)	12 (4.1%)	13 (4.1%)	33 (3.0%)
	Lay Counselor/EC	321 (65.0%)	194 (66.0%)	188 (59.3%)	703 (63.6%)

^a^At the time of this study, Swaziland national treatment guidelines recommended ART initiation at CD4 < 350 cells/μl.

^b^Patient linkage, retention, and follow-up in HIV care standard operating procedures, Swaziland National AIDS Programme, 2012.

^c^More than one cadre could be responsible for calling clients who defaulted from their first or subsequent appointment to the HIV facility, in accordance with the Linkage SOP.
